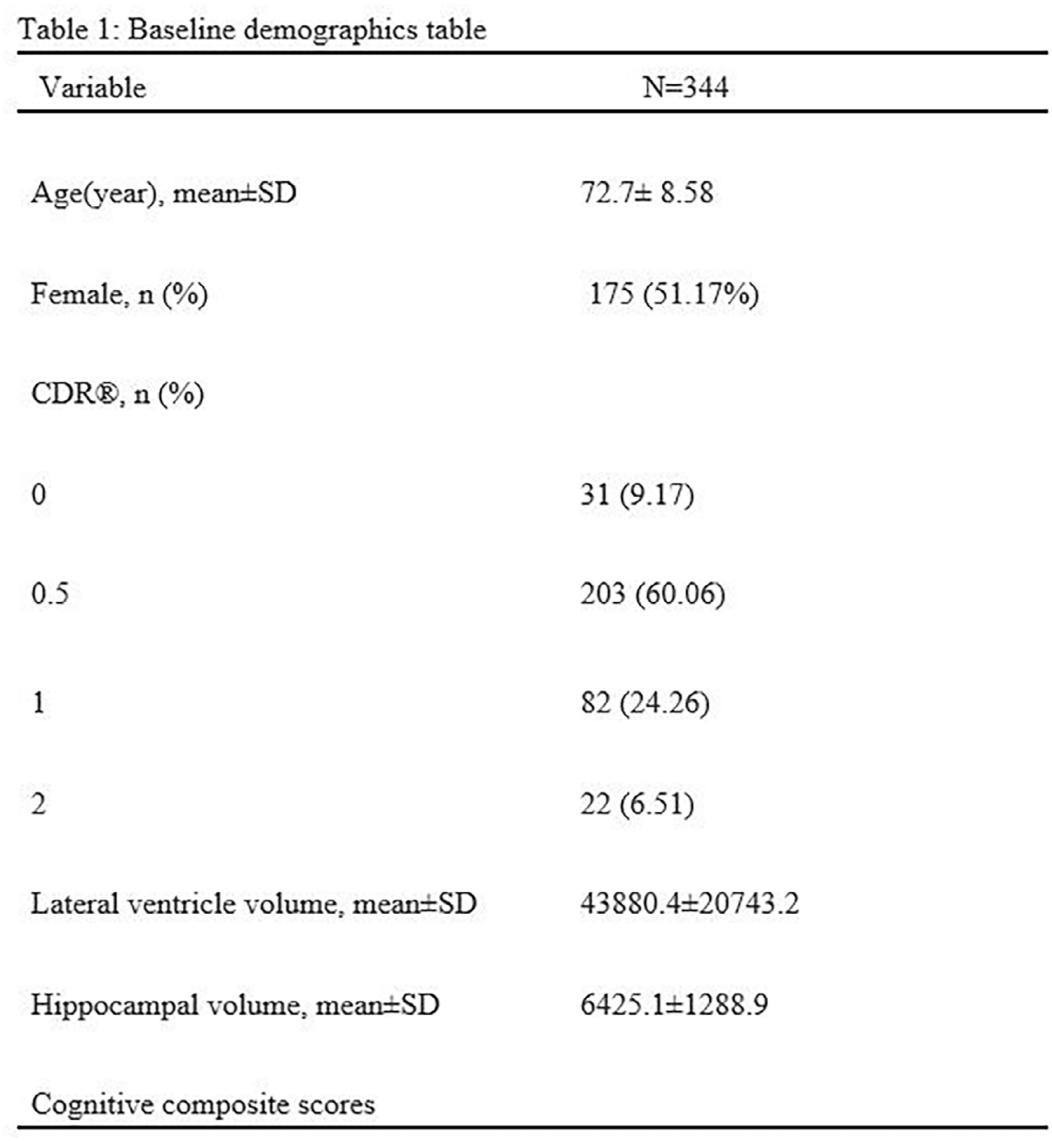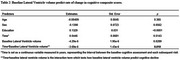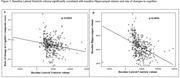# Baseline Lateral Ventricle Volume Predict Cognitive Decline in a clinical cohort

**DOI:** 10.1002/alz70856_105116

**Published:** 2026-01-07

**Authors:** Gengsheng Chen, Nicole S. McKay, Nelly Joseph‐Mathurin, Parinaz Massoumzadeh, Brian A. Gordon, Jingxia Liu, Pamela J. LaMontagne, Sarah J. Keefe, Suzanne E. Schindler, Jason J. Hassenstab, Carlos Cruchaga, John C. Morris, Tammie L.S. Benzinger

**Affiliations:** ^1^ Washington University in St. Louis, School of Medicine, St. Louis, MO, USA; ^2^ Washington University School of Medicine, St. Louis, MO, USA; ^3^ Washington University in St. Louis School of Medicine, St. Louis, MO, USA; ^4^ Washington University in St. Louis, St. Louis, MO, USA

## Abstract

**Background:**

Biomarkers are essential for the early detection of Alzheimer disease (AD) to develop effective treatments. As neurons die, hippocampal shrinkage and lateral ventricle expansion occur. Measuring hippocampal volume is challenging due to its small size, while lateral ventricle volume is easier to quantify. Our previous research showed that baseline and longitudinal changes in lateral ventricle volume predicted cognitive decline in a research cohort. However, its utility as a biomarker in clinical populations remains underexplored. This study investigated whether lateral ventricle volume predicts cognitive decline in clinical patients with memory complaints who underwent Clinical Dementia Rating (CDR©), neuropsychometric, cerebrospinal fluid (CSF) and imaging assessments between 1990 and 2018.

**Method:**

This study included 344 participants from the OASIS4 clinical cohort (https://sites.wustl.edu/oasisbrains/home/oasis‐4/) who had at least two cognitive assessments and corresponding MRI scans. Lateral ventricle and hippocampal volumes were extracted using FreeSurfer (5.3) software and adjusted for intracranial volume. Cognitive performance was measured using a composite score based on z‐scores from Verbal Fluency, Trailmaking B Seconds, and Digit Symbol Errors—tests commonly used to study early AD‐related changes. A random coefficient model examined whether baseline lateral ventricle volume predicted cognitive decline, controlling for age, sex, and education. Linear regression analyzed correlations between baseline hippocampal volume and baseline lateral ventricle volume, and the relationship between baseline lateral ventricle volume and changes in cognitive composite scores.

**Result:**

Demographic characteristics of the participants at baseline were presented in Table 1. Baseline lateral ventricle volume was a significant predictor of the cognitive decline as measured by the cognitive composite scores (Table 2, *p* =  0.01). Additionally, baseline lateral ventricles volume is significantly correlated with the rate of changes in cognitive composite scores (Figure 2A, R^2^=0.0368, *p* = 0.0003) and baseline hippocampal volume (Figure 2B, R^2^ = 0.1631, *p* < 0.0001).

**Conclusion:**

These findings suggest that the lateral ventricle volume may be used as a robust and easily measurable imaging biomarker in clinical setting. Its significant predictive value for cognitive decline, along with its strong association with hippocampal volume in a clinical cohort, highlights its potential for monitoring AD progression. This translates our research findings into real‐world populations.